# Was retrospective change measurement conducted with Covid-19 containment inconsistent? Comparing prospective and retrospective change measures using data from a national survey on substance use and addictive behaviors

**DOI:** 10.1371/journal.pone.0286597

**Published:** 2023-06-02

**Authors:** Marc Dupuis, Joseph Studer, Matthias Wicki, Simon Marmet, Gerhard Gmel

**Affiliations:** 1 Institute of Primary Health Care (BIHAM), University of Bern, Bern, Switzerland; 2 Service of Addiction Medicine, Department of Psychiatry, Lausanne University Hospital and University of Lausanne, Lausanne, Switzerland; 3 Service of Adult Psychiatry North-West, Department of Psychiatry, Lausanne University Hospital and University of Lausanne, Lausanne, Switzerland; 4 Institute for Research, Development and Evaluation, Bern University of Teacher Education, Bern, Switzerland; 5 School of Social Work, University of Applied Sciences Northwestern Switzerland, Olten, Switzerland; 6 Addiction Switzerland, Lausanne, Lausanne, Switzerland; 7 Centre for Addiction and Mental Health, Toronto, Ontario, Canada; 8 University of the West of England, Frenchay Campus, Coldharbour Lane, United Kingdom; Center for Primary Care and Public Health: Unisante, SWITZERLAND

## Abstract

Single-measurement-point data collection to assess change has increased with studies assessing the impact of the Covid-19 pandemic and of its containment, despite evidence of its lack of validity. Retrospective change is not equivalent to change in repeated self-reported measures giving raise to questions about the validity of the former. This paper purports to investigate inconsistencies between change measures by confronting retrospective change to information from longitudinally self-reported measures from the C-SURF cohort study. The study sample consists of 2,279 young men who participated in C-SURF between 2020 and 2021, and completed between May and June 2021 a survey covering change in alcohol, cigarette, cannabis and other addictive behaviors related to the pandemic. The aforementioned behaviors were assessed longitudinally at two time points using self-reports, and retrospective change since the onset of the Covid-19 crisis was also assessed at the second measurement time. Information from both prospective and retrospective change measures were confronted to identify inconsistent information for each behavior. Additionally, multiple logistic regressions were performed to assess associations between socioeconomic status, impulsivity, depression, and different indicators of motivation to complete the study and inconsistency between both measures for each behavior of interest. Importantly, inconsistent information in at least one of the investigated behaviors was found in about 90% of the participants. Small associations were found between inconsistency and different factors with a consistent effect of impulsivity. In the absence of evidence of the validity of retrospective change measures, studies relying on retrospective change should be interpreted with caution.

## Introduction

Retrospectively, it is quite clear now that no government, no health system, no citizen was really prepared to face the Covid-19 outbreak in March 2020 nor its ongoing consequences. Neither were the scientists from most of fields. The pandemic representing a never-known-so-far phenomenon, thousands of research groups all over the world saw the appealing opportunity to bring a major contribution to the knowledge of the Covid-19 and its impact. Research on Covid-19 or only taking it into account already represents millions of available manuscripts. The very question is: how much high-quality research is there among all the published work? Indeed, the gap to be filled plunged the scientific world into a race to Covid-19 publications. In their eagerness to provide insights quickly, many researchers were willing to make compromises regarding the quality of their data collection techniques. This led in the exponentiation of both data of little quality and findings of limited interest, even in high-impact journals [1]. Some authors from different fields called the phenomenon a “paperdemic” [2, 3] or “panic publishing” [4, 5].

Despite not being a Covid-19-related study, the present paper focuses on the legitimacy of one type of measurement overused during the pandemic: retrospective measurement of change. Despite various wordings (e.g., “perceived change”, “subjective change”, “self-reported change”, “retrospective change”), subjective measurement of change consists of the very same thing: the subjective assessment of change based on one sole self-report collectible in cross-sectional research. It is thus used in the absence of separate measures to identify change over time. For clarity purpose, *retrospective change* will refer in the present paper to self-reported change measured at one time point (i.e., during the crisis only), whereas *prospective change* will refer to changes between successive self-reports at two different time points (i.e., before and during the crisis).

In the specific context of the Covid-19 pandemic, retrospective change was merely used in research started consequent to the first outbreak and commonly not including prior sampling nor data collection. It was used as a proxy for change across time based on longitudinal measures, which relies on the double assumption of its validity (convergent conclusions with other measures) and reliability (same conclusions with the same measure used twice).

Several concerns have been formulated against research on change started after the Covid-19 containment came into force. First, most of studies were based on convenience or snowball sampling according to Mohler-Kuo and colleagues [6], and strong evidence of participation bias was found in online survey research conducted during the lockdown [7]. In line, participation bias and low-quality data were prevalent using online surveys [8]. Studies measuring change using self-reports and based on post-outbreak sampling were also more likely to show little representativeness [6–8], making their results unlikely to be generalizable. These issues represent important overall limitations to be mentioned, though none is the question under study.

More important, research using retrospective change measures relies on the assumption that self-reported change is equivalent to prospective change. However, the equivalence between change measurement methods is hardly assumable, especially cognitively speaking. Whereas successive prospective self-reports on present statuses cover recent representative behaviors at the measurement time, retrospective change requires the participants to recall prior statuses correctly to result in a correct perception of change. Retrospective change is thus by-design more exposed to recall bias, leading to more inaccurate or even incorrect information [9]. Moreover, generally with a longer recall time come higher risks of under- or overestimating quantity and frequency of a given substance consumption, as shown for alcohol drinking [10, 11] or cigarette smoking [12].

Retrospective change has shown little validity in different fields [13–17]. In health research, retrospective change showed variable inconsistency proportions depending on the behavior of interest [18, 19] and on the population of interest, even when measuring short-term change [15]. Moreover, even studies assessing the impact of the pandemic containment acknowledge the high probability of recall bias [20, 21]. The existing literature consists of some evidences of the lack of validity of retrospective change measures [13–19], but also lacks works demonstrating the validity of such measures.

The present study represents a simple empirical confrontation of information between prospective and retrospective change measures in order, first, to quantify the proportion of inconsistency between the latter and the last, second, to analyze the relations between flagged inconsistencies and some potential predictors of inconsistent responding, namely impulsivity and depression, and likely motivational factors. Depression consists of possible memory losses and inability to concentrate, and is likely to increase recall bias [22]; moreover, depression is also associated with a decreased motivation to complete surveys [23]. Impulsivity is also likely to increase inattentive responding, and potentially recall bias too. Higher impulsivity scores are associated with attention deficit [24], which is also likely to increase inconsistent responding.

Importantly, the aim of the study is to test whether prospective and retrospective change measures lead to divergent information (demonstrating thus that the assumption of their equivalence is questionable), not to identify which of both is the most valid.

## Material and methods

### Study design and participants

This study was based on the Cohort Study on Substance-Use Risk Factors (C-SURF), a longitudinal cohort study conducted from 2010 to 2020 in Switzerland, whose participants were recontacted for a follow-up on early Covid-19 containment impact. C-SURF participants were first contacted in 2010–2011 during the assessment of their aptitude for military or civil service. They were recruited at three of the six national military recruitment centers (in Lausanne, Windisch and Mels), which together cover 21 of Switzerland’s 26 areas (cantons) and two of the three main official languages in Switzerland (French and German). In Switzerland, men with the Swiss nationality have the obligation to participate in the assessment of their aptitude for military or civil service after their 17^th^ year of age. Because of this compulsory procedure, military centers were used for meeting and recruiting participants in a representative way. The study was independent of the army [25]. C-SURF participants were included in the study cohort regardless of their later enrollment for military or civil service or of their inaptitude for service. They were by-design about the same age, were all men with the Swiss nationality.

The study consisted of four waves in which participants could chose to complete questionnaires on their alcohol and substance use, and other factors either online or paper-pencil. At each wave, the questionnaire length was of about 50 to 60 pages, thus participants were rewarded with vouchers. The participants could complete the questionnaires either in French or in German and due to eligibility criteria for the military obligations, they had spoken one of these national languages for at least 10 years at conscription. The last wave took place from April 2019 to September 2020. This last wave is considered as the baseline of the present study and a total 5,367 completed it. Of them, 4,407 participants completed the study online and before February 15^th^, making their participation status and the date of study completion clear when the Covid-19 outbreak occurred. They were considered eligible for a short follow-up study about changes related to the Covid-19 situation. The 960 other participants were not included in this follow-up because they had either completed the study using paper-pencil questionnaires (temporarily unavailable due to lockdown) or not completed the fourth wave questionnaire already (which was normal given their later recruitment and participation in the first, second and third waves).

**Fig 1:**
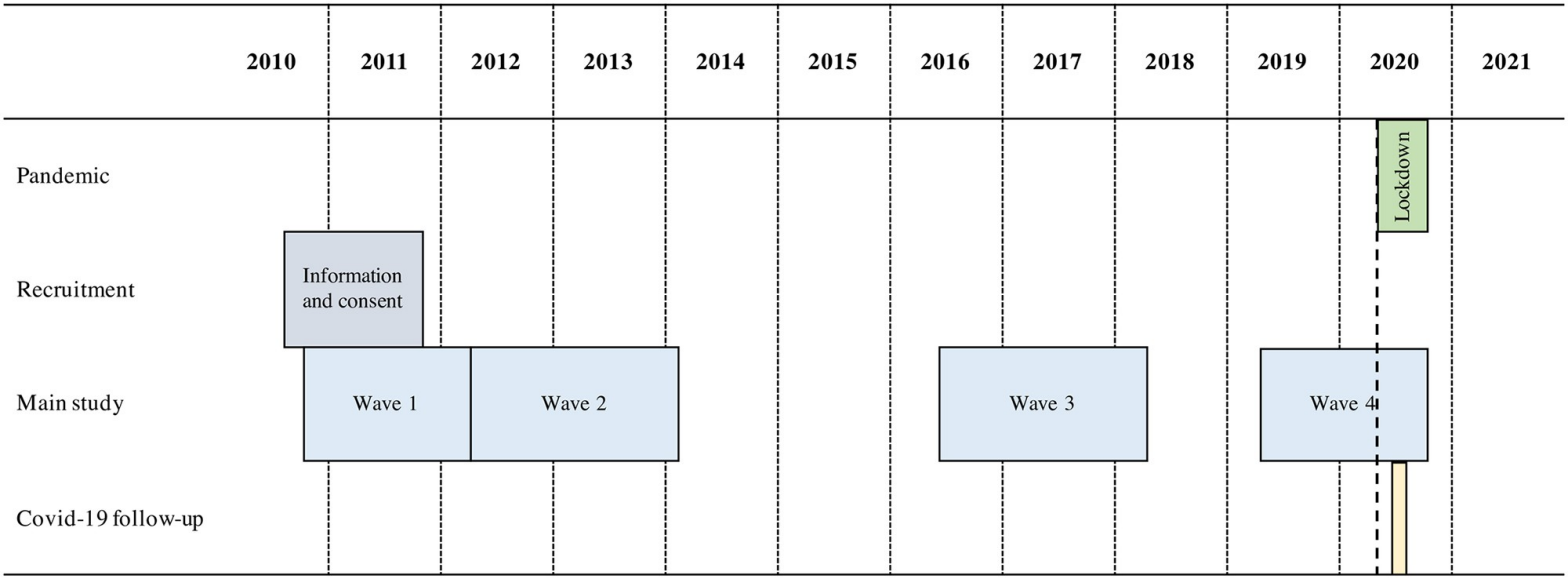
Timeline of the study.

In May 2020, the 4,407 participants were invited to complete a new online and much shorter questionnaire on the early impact of the pandemic and its containment. The invitation was sent on May 13^th^, then, reminders were sent on May 19^th^ and 26^th^ to participants who had not yet completed the survey. Data collection ended on June 8^th^. The questionnaire length was equivalent to 15 paper pages and could be completed in about 20 minutes (a median completion time of 16 minutes was measured). Technically speaking, the survey was conducted on *LimeSurvey*, was completable only on email invitation (and, as mentioned, independent from any financial reward), which represented the best protective factors against the presence of malicious software (bots) taking the survey, according to the literature so far [26, 27]. For 18 participants whose email addresses were incorrect or not up to date, they were recontacted first by SMS and received personal invitation links. Fig 1 provides an overview of the timeline.

A total of 2,545 participants (57.7% of the available participants) started filling out the questionnaires and 2,279 (89.5%) completed the survey. The present study is based on these 2,279 participants who fully completed the “Covid-19” follow-up study. A detailed description of the Covid-19 sample and its characteristics has been published already [28].

The Lausanne University Medical School’s Clinical Research Ethics Committee, and then the Human Research Ethics Committee of the Canton of Vaud approved the research protocols for the different waves of the study, including the Covid-19 follow-up (protocol 15/07, PB_2018–00296). Data and additional information are available online on Zenodo [29].

### Measurements

#### Prospective change

Several consumption or potentially addictive behaviors were assessed at both baseline (fourth wave C-SURF) and follow-up (Covid-19 follow-up): binge drinking, cigarette, cannabis use, video gaming, TV series use, and internet pornography use. At baseline, participants were asked to describe the typical frequency (or both quantity and frequency) of each behavior for the past 12 months, whereas at follow-up they had to describe the representative frequency (or both quantity and frequency) of each behavior since the Covid-19 containment, that is to say for about the past two months. Despite the difference in the duration of the covered periods, the questions were investigated in a consistent way at both assessments.

Concerning the metrics, the different questions and possible answers are presented in S1 Table. Briefly, binge drinking and cannabis use was measured only based on their frequency, for the past 12 months at baseline and from the beginning of the pandemic containment measures at follow-up. Cigarette use, video gaming and TV series and movie use was measured in weekly number of cigarettes and in weekly time spent in front of a screen, respectively. Finally, internet pornography use was measured in monthly time spent on any online pornographic content. For these four last behaviors, status and subsequent change were estimated based on a quantity-frequency product.

**Fig 2:**
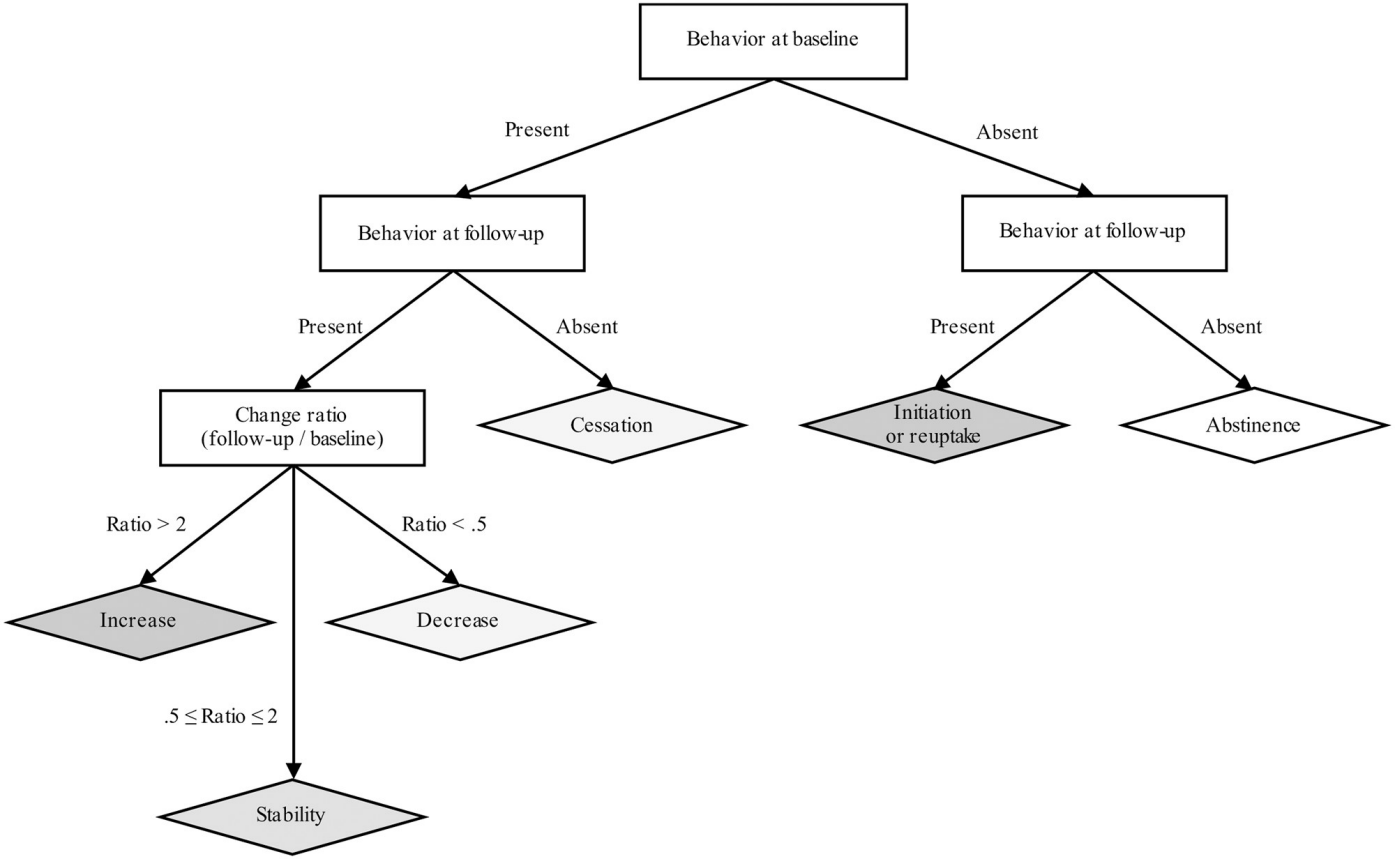
Flowchart for the categorization of prospective change in self-reported measures. Nota bene: the legend corresponds to changes measured using both frequency and quantity (i.e., smoking, video gaming, internet pornography use, and TV series and movie watching). The decisional procedure is the same for binge drinking and cannabis use that are measured only in frequency, though no ratio calculation is needed to differentiate increase, decrease and stability.

Prospective change over time was then categorized using six categories, namely: cessation (when the behavior was present at baseline and absent at follow-up), decrease, stability, or increase, initiation/reuptake (when the behavior was absent at baseline and present at follow-up), and abstinence when the behavior of interest was absent throughout the study. When the behavior was present at both baseline and follow-up, change was expressed by the ratio between follow-up and baseline measures; ratios ranging from ½ to 2 were assumed as corresponding to stability, whereas values below ½ represented a decrease and values above 2 represented an increase (Fig 2). For binge drinking and cannabis use that were assessed using the same ordinal frequency measures at each wave, change in self-reports was simply defined based on moves on the ordinal frequency metrics.

#### Retrospective change

In the follow-up survey, participants were asked to report how they changed regarding each aforementioned behavior. The questions and the possible answers were: “Since the beginning of the Covid-19 containment, my [*behavior*]: 1) has decreased; 2) has not changed; 3) has increased; 4) does not apply, I don’t [*experience the behavior of interest*].” Importantly, this last option did mean that the participants were not experiencing the behavior of interest since/during the Covid-19 containment, which did not systematically mean that they never experienced it prior to the pandemic, especially at baseline.

#### Inconsistency between prospective and retrospective change

Prospective change was compared to retrospective change. The identification of inconsistency was designed in a conservative way. Thereby, information was presumed consistent unless both change measures on a same behavior belonged to clearly incompatible categories (Table 1). Seemingly incompatible combinations were flagged as inconsistencies, which reflected, two kinds of inconsistencies:

- Inconsistencies regarding change corresponded to cases where both prospective and retrospective changes were consistent about the fact that the behavior had been experienced, and inconsistent about how its frequency (or quantity and frequency) changed over time. Importantly, when participants indicated either an increase or a decrease in the retrospective change questions and were considered stable over time based on their self-reported statuses, their responses were considered inconsistent only if change measures were diverging (e.g., increase as the retrospective change and a small decrease assimilable as stability as the result of the prospective). Thus, a ratio of about 1 to 2 was considered consistent when one reported an increase in a given behavior, and a ratio of ½ to 1 was considered consistent when one reported a decrease.
- Contradictions regarding the presence of the behavior corresponded to cases where the behavior was reported present at the follow-up in the prospective question and absent (not applicable) in the retrospective change question. This situation was considered possible in regard with the “does not apply, I don’t…” option in the retrospective change questions.

**Table 1:** Contingency table for identifying inconsistency in change measures.

	Prospective change between baseline and follow-up					
	Abstinence	Cessation	Decrease	Stability	Increase	Initiation or reuptake
**Retrospective change**						
Not applicable (absent)	✓	✓				
Decrease		✓	✓	(✓)		
Stability	✓			✓		
Increase				(✓)	✓	✓

✓: Consistency assumed between both information on change for a behavior of interest

(✓): Consistency assumed if prospective and retrospective change have the same direction and a change ratio of .5 to 2

As indicated in Table 1, several categories of retrospective change could be considered compatible with the corresponding measures of prospective change and thus assumed as “consistent”. Some of them were not intuitive. For example, if a participant had completely ceased smoking cannabis according to prospective measures, he could answer “not applicable” or “decrease”. For someone stating in the retrospective change question that his cannabis use increased and being considered stable based on prospective measures, the answers are considered consistent if the increase was smaller than twice the initial quantity.

#### Factors explaining inconsistency

Two kinds of factors potentially associated with inconsistency were taken into account, namely factors related with lower attention and motivation, which included depression and impulsivity, and two other proxies for participants interest in the study. The presence of major depressive disorder at both baseline and follow-up was assessed using the Major Depression Inventory (MDI), a screening instrument from WHO [30–32]. The MDI focuses on depressive mood experienced during the past 2 weeks. It is a 12-item questionnaire for which available responses are on a six-point scale, from never (coded 0) to all the time (5); values of the total score above 21 are indicating the presence of a major depressive episode [31].

Impulsivity was assessed at baseline using the total score of the short UPPS-P, a 20-item scale of impulsivity expressed in a 4-point Likert-type scale [33]. Though the short UPPS-P consists of five factors, they can be regrouped in a higher-order factor covering general impulsivity, which was the case for the present study. Unlike depression, impulsivity is considered as a stable factor among adults and was thus not reassessed at follow-up.

Additionally, two indicators at follow-up were also taken into account. First, participants were asked whether they would like to receive feedback regarding the Covid-19 part of the study. Second, the number of reminders sent to participants were accounted for. Each information is reflecting participants’ interest for the study at follow-up and their motivation to complete it; thus, respondents completing a study with little motivation are at higher risk to respond with less care and to bypass some sections by reporting that the question was not applicable because they did not experience the behavior [34, 35]. People who declined receiving feedback and who were sent reminders were thus considered likely to provide less consistent information.

Finally, socioeconomic status has also been shown a linear association with consistency in responding to surveys [36]. It was reported at baseline using a 7-point Likert-type scale ranging from 1) much lower than other people; to 7) much higher.

### Statistical analyses

Multiple logistic regressions were performed to measure the associations between the aforementioned factors (SES, depression, impulsivity and motivation) and inconsistency in the different sections investigated (i.e., distinct questionnaires covering participant experience of the Covid-19 crisis and containment; alcohol use since then; cigarette smoking; cannabis use; etc.). The same regression model was conducted to estimate the association between these factors and the presence of inconsistency in any section of the survey. The associations were reported in terms of odds ratios (OR) and 95% confidence intervals (CI). In the regression model, multicollinearity between socioeconomic status, depression and the number of reminders received was estimated using the variance inflation factor (VIF). VIF values of about 1 indicate that multicollinearity is absent and thresholds of 10 or higher are largely used [37]. Statistical analyses were performed using SPSS 26, and pairwise deletion was applied in case of missing values covering one of the variables of interest for 66 of the 2,279 participants (2.9%).

## Results

First, resulting from the identification of inconsistency across measures of change for the respective behaviors, 2,060 (90.5%) the participants showed at least one inconsistency. As presented in Table 2, more than 10% of inconsistency was found in each of the different sections (except of cannabis use), about 33% regarding binge drinking and about 40% to 55% video gaming, internet pornography use, TV series and movie use. Inconsistencies mostly consisted of cases in which participants reported consuming a given substance or experiencing a given behavior at both measurement times and had a different perception of change. Noteworthy, inconsistency could regard participants who reported increases or decreases in their behaviors, but it concerned substantial numbers of participants who reported stability in their behaviors as well. The least frequent case of inconsistency concerned reporting that the change question was not applicable while reporting non-null values in the follow-up questions. It represented 1.6% to 7.8% of the inconsistencies identified in the sections.

**Table 2:** Sample information.

Variable							
Prospective change	Abstinence	Cessation	Decrease	Stability	Increase	Initiation or reuptake	Total^a^
Binge drinking	465 (20.4%)	766 (33.7%)	239 (10.5%)	541 (23.8%)	209 (9.2%)	56 (2.5%)	2,276
Cigarette smoking	1,399 (61.4%)	252 (11.1%)	120 (5.3%)	379 (16.6%)	87 (3.8%)	42 (1.8%)	2,279
Cannabis use	1,649 (72.4%)	311 (13.6%)	29 (1.3%)	192 (8.4%)	59 (2.6%)	39 (1.7%)	2,279
Video games	348 (15.3%)	288 (12.6%)	742 (32.6%)	529 (23.2%)	214 (9.4%)	157 (6.9%)	2,278
Internet pornography use	319 (14.1%)	337 (14.9%)	403 (17.8%)	866 (38.3%)	252 (5.7%)	87 (3.8%)	2,264
TV series and movies watching	88 (3.9%)	146 (6.4%)	313 (13.8%)	765 (33.6%)	831 (36.5%)	133 (5.8%)	2,276
**Retrospective change**	**Not applicable**		**Decrease**	**Stability**	**Increase**		
Binge drinking	727 (32.0%)		827 (36.4%)	568 (25.0%)	151 (6.6%)		2,273
Cigarette use	1,593 (70.1%)		156 (6.9%)	326 (14.3%)	197 (8.6%)		2,272
Cannabis smoking	1,852 (81.5%)		69 (3.0%)	227 (10.0%)	123 (5.4%)		2,271
Video games	575 (25.3%)		94 (4.1%)	558 (24.5%)	1,047 (46.0%)		2,274
Internet pornography use	473 (20.8%)		270 (11.9%)	1,110 (48.8%)	423 (18.6%)		2,276
TV series and movies watching	177 (7.8%)		110 (4.8%)	756 (33.2%)	1,233 (54.1%)		2,276
**Inconsistency by retrospective change**	**Not applicable**		**Decrease**	**Stability**	**Increase**		
Binge drinking	56 (7.6%)		295 (39.9%)	301 (40.7%)	87 (11.8%)		739 (32.5%)
Cigarette smoking	21 (7.6%)		50 (18.1%)	89 (32.2%)	116 (42.0%)		276 (12.1%)
Cannabis use	15 (7.8%)		30 (15.5%)	82 (42.5%)	66 (34.2%)		193 (8.5%)
Video games	40 (3.2%)		58 (4.7%)	322 (26.0%)	819 (66.1%)		1239 (54.5%)
Internet pornography use	16 (1.6%)		157 (15.8%)	517 (52.1%)	302 (30.4%)		992 (43.9%)
TV series and movies watching	27 (2.7%)		37 (3.7%)	465 (46.5%)	470 (47.0%)		999 (43.9%)
Any inconsistency among sections							2,060 (90.5%)
**Other variables** ^b^							
Socioeconomic status [1–7]							4.02 ± 1.27
Impulsivity (before Covid-19) [20–80]							43.55 ± 5.55
Major depression before Covid-19							200 (8.8%)
Major depression during Covid-19							159 (7.0%)
Completed the survey before any reminder							1,176 (51.6%)
Completed the survey after one reminder							640 (28.1%)
Completed the survey after two reminders							417 (18.3%)
Declined receiving feedback							1,178 (51.7%)

^**a**^ The percentages in *Total* indicate the proportion of flagged inconsistency. Elsewhere, they correspond to the proportions for a given status/answer (n divided by the total).

^b^ The mean and the standard deviation are provided for numerical variables.

Second, the different logistic regression models resulted in various small significant effects of the identified factors (Table 3). Impulsivity was significantly associated with more inconsistency regarding every of the behavior of interest, except TV series and movie watching (OR ≅ 1.05). Additionally, having received two reminders before study completion was associated with more inconsistency regarding binge-drinking (OR = 1.36). For inconsistency in binge drinking, video gaming and internet pornography use sections, depression during Covid-19 crisis had also an effect (OR = 0.63, OR = 1.46 and OR = 1.47, respectively). In the cannabis section, the socioeconomic status was associated with less inconsistency (OR = 0.75). In both video game and internet pornography use sections, there were no other significant factor explaining inconsistency, except impulsivity. Impulsivity was also the only significant factor in the model measuring the association with inconsistency in almost any section (or for any of the corresponding behaviors). Noteworthy, the VIF estimates were of 1.10 and 1.06 for depression at baseline and follow-up, of 1.00 for the number of reminders and of 1.04 for socioeconomic status, which implied an almost total absence of collinearity between factors.

**Table 3:** Logistic regressions models for inconsistency between retrospective and prospective change measures.

Study section^a^	Factor	*OR*	95% CI
Binge drinking (n = 2,221)	Socioeconomic status	1.04	0.97–1.12
	Impulsivity	1.04***	1.02–1.06
	Major depression before Covid-19	0.93	0.66–1.31
	Major depression during Covid-19	0.63*	0.43–0.94
	Completed the survey before any reminder	1	
	Completed the survey after one reminder	1.06	0.86–1.3
	Completed the survey after two reminders	1.36**	1.08–1.73
	Declined receiving feedback	1.08	0.9–1.29
Cigarette smoking (n = 2,222)	Socioeconomic status	0.90	0.82–1.00
	Impulsivity	1.03***	1.01–1.06
	Major depression before Covid-19	1.04	0.66–1.63
	Major depression during Covid-19	1.44	0.91–2.29
	Completed the survey before any reminder	1	
	Completed the survey after one reminder	1.00	0.74–1.35
	Completed the survey after two reminders	1.11	0.79–1.55
	Declined receiving feedback	0.99	0.77–1.29
Cannabis use (n = 2,221)	Socioeconomic status	0.75***	0.67–0.85
	Impulsivity	1.06***	1.03–1.09
	Major depression before Covid-19	0.74	0.43–1.30
	Major depression during Covid-19	1.14	0.64–2.04
	Completed the survey before any reminder	1	
	Completed the survey after one reminder	1.33	0.94–1.88
	Completed the survey after two reminders	1.15	0.77–1.73
	Declined receiving feedback	0.89	0.66–1.21
Video gaming (n = 2,223)	Socioeconomic status	0.96	0.90–1.03
	Impulsivity	1.02*	1.00–1.03
	Major depression before Covid-19	0.96	0.69–1.32
	Major depression during Covid-19	1.46*	1.02–2.07
	Completed the survey before any reminder	1	
	Completed the survey after one reminder	1.09	0.90–1.32
	Completed the survey after two reminders	0.98	0.78–1.23
	Declined receiving feedback	0.89	0.75–1.05
Internet pornography use (n = 2,213)	Socioeconomic status	0.97	0.91–1.04
	Impulsivity	1.03***	1.01–1.05
	Major depression before Covid-19	1.05	0.76–1.44
	Major depression during Covid-19	1.47*	1.04–2.07
	Completed the survey before any reminder	1	
	Completed the survey after one reminder	1.11	0.91–1.35
	Completed the survey after two reminders	0.90	0.72–1.13
	Declined receiving feedback	0.98	0.83–1.16
TV series and movies watching (n = 2,226)	Socioeconomic status	0.96	0.90–1.03
	Impulsivity	1.01	0.99–1.02
	Major depression before Covid-19	1.08	0.78–1.48
	Major depression during Covid-19	0.78	0.55–1.11
	Completed the survey before any reminder	1	
	Completed the survey after one reminder	1.11	0.92–1.35
	Completed the survey after two reminders	0.86	0.68–1.08
	Declined receiving feedback	1.11	0.94–1.32
Any inconsistency (n = 2,213)	Socioeconomic status	0.90	0.80–1.01
	Impulsivity	1.04**	1.01–1.06
	Major depression before Covid-19	1.00	0.56–1.78
	Major depression during Covid-19	1.27	0.66–2.44
	Completed the survey before any reminder	1	
	Completed the survey after one reminder	1.15	0.82–1.61
	Completed the survey after two reminders	1.04	0.71–1.53
	Declined receiving feedback	0.99	0.74–1.31

*p < 0.05,

**p < 0.01,

***p < 0.001

^a^ The differences in sample sizes between sections are due to missing values in key variables.

## Discussion

The aim of the present study was to investigate how inconsistent subjective measurements of change were. One of the paper’s major findings is certainly the high degree of inconsistency between answers in the questionnaires: among more than 2,200 respondents, about 30% of them showed inconsistency regarding binge drinking, about 55% regarding video gaming, and about 44% regarding internet pornography use and regarding TV series watching. In comparison, inconsistency was much less frequent regarding cigarette smoking and cannabis use (i.e., about 10%), likely because only a third of the participants had a consumption that they could actually under- or overestimate. Cumulatively, 90.5% showed inconsistency between prospective and retrospective changes for at least one behavior.

Concerning the regression models, the present study tested the associations between inconsistency and both attentional and motivational factors. The significant associations between inconsistency and indicators of lower motivation to complete the study (the reminders and the refusal to receive a feedback) could represent evidence of the contribution of motivation to participate in such surveys. The proxies for motivation to complete the study were, however, rarely significantly associated with inconsistency in the models, and having declined receiving feedback on the Covid-19 follow-up was significant in neither of them. On the other hand, attentional factors were significantly associated with inconsistency in each model. Despite the small OR found (about 1.05), impulsivity was measured on a 60-point large score, making its actual role far from negligible (i.e., accordingly, the OR between participants with differences of 20, 30 and 40 points would be of about 2.65, 4.32 and 7.04, respectively).

Interestingly, depression was significant only at follow-up, also suggesting that inconsistency occurs rather at follow-up than shared between baseline and follow-up. Despite the well-known risk of increased recall bias among depressive participants, they were actually more consistent in their responses than non-depressive participants. Depression during the Covid-19 crisis could have played a role as a motivation to participate in the follow-up that was focusing on changes consequent to the situation; if so, the motivational role of depression represented a stronger protective factor than the higher rate of recall bias in depressive individuals.

Measuring the same behavior twice using the same self-administered instrument longitudinally is far from easy and quick, because it requires to assess respondents later and to deal with participation and attrition bias. It represents so far the standards to measure change in health behaviors correctly [12, 38, 39]. In contrast, retrospective measurement of change has shown evidence of its lack of reliability and high risk of recall bias [9]. Metaphorically speaking, inferring conclusions based on information measured only once and solely retrospectively may be seen as a muddy shortcut taken by some researchers. No matter the intentions nor the material conditions underlying the methodological choice, the most important question is whether the shortcut and the long but safe road bring the walkers to the very same place. In other words, both measurement techniques should lead to convergent information. The present study highlighted that it was not the case for a large majority of participants. The study showed that both measurement procedures did frequently not converge. The methods were by design able to highlight the existence of differences between both change measurement techniques, indicating that at least one of them is more biased, but not to bring decisive information on which of them is biased; additionally, both rely on self-reports, which implies subjectivity in assessing health behaviors. Nonetheless, measuring change in successive self-reported statuses is in line with methodological standards up to now, whereas little is known about retrospective change measurement, apart from existing claims on its lack of validity. The present findings should thus be considered as an additional warning about the probable lack of validity of retrospective change measures.

Resulting from the analyses, another finding was the significant associations between inconsistency and depression, impulsivity, and SES. The associations were small overall. However, the potential impacts of the factors may vary across studies so that they could be not negligeable at all in other studies. Not accounting for them in the future might lead to potential errors.

This study is straightforward but has also limitations to discuss. In line with the main limitations of the C-SURF study, the present findings are based on males only (women might be more consistent than men in their responses), they rely on data from Swiss male adults of about 30 years of age (M = 29.1 ± 1.3), but not of non-national citizen living in Switzerland, for instance migrants, who may be at even higher risk of providing inconsistent information due to language barriers. Only 51.7% of the available participants took part in the follow-up survey. Although, representativeness was not necessary in order to show that prospective and retrospective change measures were not converging. Moreover, the present findings depend, of course, on the threshold considered to define change *versus* stability. Considering that a ratio of 2 was large enough to represent psychologically meaningful change in a given behavior across time was arbitrary. Nevertheless, large proportions of inconsistency were found with self-reported decreases or increases in behaviors, and with self-reported stability as well. This suggested that the thresholds used was neither simply too sensitive nor too conservative. In addition, the arbitrary thresholds do not alleviate the question of respondents who reported having not experienced some behaviors in the change question despite contradictious answers elsewhere in the follow-up survey. The proportion of this specific inconsistency was rather small (1.6% to 7.8%). This most probably suggests the presence of a strategy to complete the survey quickly by bypassing additional questions, in line with a loss of motivation.

Throughout the entire duration of C-SURF, investigation focused on several sensitive topics. In the present study, this includes illicit drug use and pornography use. Social desirability might have influenced participants answer from the very beginning. However, it is unlikely that differences in socially desirable responding may emerge across time, making this potential bias not very concerning. Furthermore, given that 66% of the respondents admitted an increase in time spent using pornography, it suggests that they remained honest in responding.

One last point to be discussed is the fact that 1,862 C-SURF participants did not took part to follow-up. Though their absence would represent a significant limitation for many studies aiming to describe populations in a representative way, it is rather a strength for this situation. Participants who did not participate in the follow-up were more likely to be prone to response bias such as inattentive responding. Thus, the present study highlights that inconsistencies between subjective measures of change occur among compliant respondents.

## Conclusion

Since the beginning of the pandemic, several authors have warned the scientific community about the insufficient quality of Covid-19 studies in every field [1, 4, 5]. Though similar crises might hopefully not occur soon, one lesson that may be learned concerns methodological strategies to be chosen next times. Shoib and his colleagues [5] stated in 2021 that there had been thousands of surveys related to Covid-19 crisis and mental health. Overall, they wondered if only half of them had gone through any ethical process at all. They were probably right: because cross-sectional questions on change permit fully anonymized data collection, surveys relying on such a design are likely neither to fall within the scope of laws on human research nor to need the approval of an ethics committee. This represents a shortcut route.

Longitudinal data collection has a cost, but it is resulting in higher-level evidence for reasons. Given that research consequent to the pandemic was prone to sampling issues and that there is little evidence supporting the validity of retrospective measurement of change, studies conducted only based on such a method to collect information seem of limited interest *per se*. It is clearer now that longitudinal data and cohorts from studies started and sampled before 2020 were available worldwide at this moment [6, 40–42], some of them could eventually be used to have better insight on change-related questions. Other researchers used retrospective change measures combined with sale statistics in order to mitigate the potential limitations of a study entirely relying on the latter [11]. This illustrates the possibilities to improve confidence in results by providing converging results based on different proxies.

In the absence of evidence for their validity, retrospective measures of change should be used only when justified by strong rationales, not simply for convenience reasons. They should be used with other measures and interpreted only with great caution.

## Supporting information

S1 Table Specific questions and answers for each behavior of interest. (DOCX)
