# Supplementary material for: Was retrospective change measurement conducted with Covid-19 containment inconsistent? Comparing prospective and retrospective change measures using data from a national survey on substance use and addictive behaviors
Source: PLoS One. 2023 Jun 2;18(6):e0286597. doi: 10.1371/journal.pone.0286597 (PMC10237494; doi:10.1371/journal.pone.0286597)
Supplement: S1 Table — (DOCX) [file pone.0286597.s001.docx]

**Supporting information**

**S1 Table. Specific questions and answers for each behavior of interest.**

| **Study section** | **Question / possible answers** |
| --- | --- |
| Binge drinking | **How often do you drink six or more standard drinks or more on a single occasion?** |
|  | Never |
|  | Less than once a month |
|  | Every month |
|  | Every week to every day |
| Cigarette smoking | **How often have you generally smoked cigarettes?** |
|  | Never |
|  | Once in a month or less |
|  | 2 to 3 days a month |
|  | 1 to 2 days a week |
|  | 3 to 4 days a week |
|  | 5 to 6 days a week |
|  | Every day |
|  | **On a usual day when you smoke cigarettes, how many cigarettes do you smoke?** |
|  | [Numerical value: number of cigarettes] |
| Cannabis use | **How often did you usually take “illegal” cannabis? (i.e., with more than 1% THC)** |
|  | Never |
|  | Once in a month or less |
|  | 2 to 4 times a month |
|  | 2 to 3 times per week |
|  | 4 to 5 times per week |
|  | Every day or nearly every day |

**S1 Table (continued). Specific questions and answers for each behavior of interest.**

| **Study section** | **Question / possible answers** |
| --- | --- |
| Video gaming | **How often did you play video games (online, offline, on console or on smartphone)?** |
|  | Never |
|  | A few times |
|  | 1 to 3 times a month |
|  | 1 to 2 times a week |
|  | 3 to 4 times a week |
|  | Every day or almost every day |
|  | **On a typical day on which you played video games, how long did you play on average?** |
|  | [Numerical value: hours and minutes] |
| Internet pornography use | **How many days a month do you visit pornographic web sites usually?** |
|  | [Numerical value: days a month] |
|  | **How much time do you spend on the Internet to visit pornographic websites on days when you visit pornographic websites?** |
|  | None |
|  | Almost none |
|  | < 1 hour |
|  | 1 hour to < 2 hours |
|  | 2 hour to < 3 hours |
|  | 3 hour to < 4 hours |
|  | 4 hours or more |
| TV series and movies watching | **How often did you watch TV series/movies (television, Netflix, Amazon, streaming, DVD, etc.)?** |
|  | Never |
|  | Some times |
|  | 1-3 times per month |
|  | 1-2 times per week |
|  | 3-4 times per week |
|  | Daily or almost daily |
|  | **On a typical day on which you watch TV series, for how long do you watch them on average?** |
|  | [Numerical value: hours and minutes] |
